# Exploring the health and sociodemographic characteristics of people seeking advice with claiming universal credit: a cross-sectional analysis of UK citizens advice data, 2017–2021

**DOI:** 10.1186/s12889-023-15483-4

**Published:** 2023-03-30

**Authors:** Heather Brown, Huasheng Xiang, Mandy Cheetham, Steph Morris, Marcia Gibson, Srinivasa Vittal Katikireddi, Luke Aaron Munford, David Taylor-Robinson, Hannah Finney, Victoria Bartle, Andrew J Baxter, Sophie Wickham, Peter Craig, Clare Bambra

**Affiliations:** 1 Division of Health Research, Lancaster University, Lancaster, UK; 2 Population Health Sciences Institute, Newcastle University, Newcastle, UK; 3 Nursing, Midwifery and Health, Northumbria University, Newcastle upon Tyne, UK; 4 MRC/CO Social and Public Health Sciences Unit, University of Glasgow, Glasgow, UK; 5 Health Organisation, Policy and Economics, School of Health Sciences, University of Manchester, Manchester, UK; 6 Department of Public Health, Policy and Systems, Institute of Population Health, University of Liverpool, Liverpool, Merseyside, UK; 7 Citizens Advice Newcastle, Newcastle Upon Tyne, UK; 8 Centre for Translational Research in Public Health, Newcastle upon Tyne, UK

**Keywords:** Limiting Long Term Conditions, Mental health, Sociodemographic characteristics, Universal Credit, Citizens advice, UK

## Abstract

**Background:**

The UK Department for Work and Pensions (DWP) administers Universal Credit (UC) – the main UK benefit for people in- and out-of-work. UC is being rolled out nationally from 2013 to 2024. Citizens Advice (CA) is an independent charity that provides advice and support to people making a claim for UC. The aim of this study is to understand who is seeking advice from CA when making a UC claim and how the types of people seeking advice are changing as the rollout of UC continues.

**Methods:**

Co-developed with Citizens Advice Newcastle and Citizens Advice Northumberland we performed longitudinal analysis of national data from Citizens Advice for England and Wales on the health (mental health and limiting long term conditions) and socio-demographic of 1,003,411 observations for people seeking advice with claiming UC over four financial years (2017/18 to 2020/21). We summarised population characteristics and estimated the differences between the four financial years using population-weighted t-tests. Findings were discussed with three people with lived experience of seeking advice to claim UC to help frame our interpretation and policy recommendations.

**Results:**

When comparing 2017/18 to 2018/19, there was a significantly higher proportion of people with limiting long term conditions seeking advice with claiming UC than those without (+ 2.40%, 95%CI: 1.31-3.50%). However, as the rollout continued between 2018/29 and 2019/20 (-6.75%, 95%CI: -9.62%--3.88%) and between 2019/20 and 2020/21 (-2.09%, 95%CI: -2.54%--1.64%), there were significantly higher proportions of those without a limiting long term condition seeking advice than with. When comparing 2018/19 to 2019/20 and 2019/20 to 2020/21, there was a significant increase in the proportion of self-employed compared to unemployed people seeking advice with claiming UC (5.64%, 95%CI: 3.79-7.49%) and (2.26%, 95%CI: 1.29-3.23%) respectively.

**Conclusion:**

As the rollout for UC continues, it is important to understand how changes in eligibility for UC may impact on those who need help with applying for UC. Ensuring that the advice process and application process is responsive to a range of people with different needs can help to reduce the likelihood that the process of claiming UC will exacerbate health inequalities.

**Supplementary Information:**

The online version contains supplementary material available at 10.1186/s12889-023-15483-4.

## Background

**Fig. 1:**
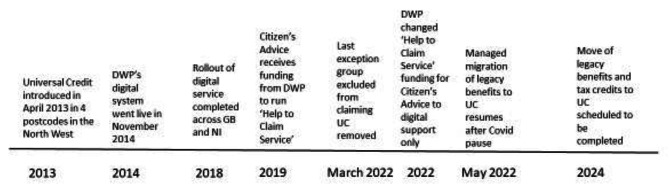
Timeline of rollout of Universal Credit across Great Britain and Northern Ireland

Health inequalities are continuing to rise in the UK [1]. In 2013, the UK Government implemented a major reform of social security with the introduction of Universal Credit (UC). UC combined six benefits and tax credits (income-based Job Seekers Allowance, income-related Employment and Support Allowance, Income Support, housing benefit for working age claimants, Child Tax Credit, and Working Tax Credit) which are known as ‘legacy benefits’ into a single payment. Figure 1 describes the rollout of UC across Great Britain and Northern Ireland. There is a growing body of evidence showing that UC is having a negative health impact on those claiming it [2–5].

Citizens Advice (CA) is an independent network of local charities, that offers free, impartial advice online, over the telephone, and in person [6]. From 2019, CA has received funding from the Department for Work and Pensions (DWP) to provide advice with the claim process for UC.

In contrast to legacy benefits and tax credits, UC is a ‘digital by default’ service [7]. The DWP acknowledges that many of its service users include vulnerable people who may have difficulty accessing the internet [7]. 92% of the UK population regularly uses the internet; however, for disabled adults this figure is 81% [8]. The DWP has taken a “test and learn approach” to UC; in other words, committing to making improvements when issues present themselves. Some issues identified include difficulties with the digital claim process, errors and delays in payments leading to financial hardship and debt [2]. A number of solutions have been identified to some of these problems, but they have not yet been implemented [9].

Because of the different routes and triggers for moving from legacy benefits to UC, there is regional variation in the proportion of households on UC compared to those receiving legacy benefits. Cuts to local government budgets from 2010 to 2020 have led to a reduction in local services, such as libraries, which have provided a point of access for digital literacy skills and access to computers [10]. Cuts to services vary by local authority so changes in provisions of these services will vary across the country. These regional differences may be an additional contributing factor to regional variations in health inequalities associated with UC.

Qualitative evidence from the North East of England suggests that many claimants struggled with the digital claim process [2]. However, nationally we do not know who may be struggling with claiming UC and thus, seeking advice from charities such as Citizens Advice and how this may be a contributing factor to poor health associated with UC [2–4].

The aim of our study is to understand who is seeking advice with claiming UC and how this may be changing over time as UC is rolled out. We hypothesise that as UC is rolled out across the country and to different groups the number of people seeking advice will increase as more people become eligible for UC. However, it is possible, if as more people are rolled out on to UC, DWP simplifies the benefit procedure, which could then reduce the number of people seeking advice with claiming. Our second hypothesis is that as UC is rolled out to people claiming different legacy benefits the composition of people seeking advice will change. Understanding if the number and composition of people seeking advice has changed as UC is rolled out is important for both the DWP who administers UC and organisations that support those with the claim process such as Citizens Advice. If the claim seeking process is particularly challenging for certain groups the stress associated with this process and potentially the financial stress associated with making mistakes may have a negative impact on health contributing to health inequalities. If there are regional variations in claim rates this may contribute to increasing regional health inequalities. This research is part of a larger mixed method evaluation of UC on mental health [5]. We utilised a unique data source from Citizens Advice on all UC claimants who sought advice across England and Wales between April 2017 and March 2021. We employed a co-production approach developing this work with Citizens Advice Newcastle and Citizens Advice Northumberland in the North East of England. Our findings can be used to develop policy in conjunction with Citizens Advice and DWP (who administer UC) to ensure there is the support and services in place to ensure that everyone claiming UC is able to access the advice that they need. Demystifying and simplifying the claim process can be an important step to reducing the negative health impacts associated with UC.

## Methods

We co-produced a longitudinal analysis of national data from Citizens Advice service and developed recommendations for policy and practice through involvement of service users.

### Co-Development and user involvement

We co-produced the research with staff from Citizens Advice Northumberland and Citizens Advice Newcastle, who helped us to shape the research questions and to inform the analysis, in order to better understand their local clients and how their needs have been changing over time.

When the project commenced in May 2021, we formed an advisory group, which included the research teams and advisors from Citizens Advice local offices in Gateshead, Newcastle, Northumberland, and Sunderland. We held three meetings with this advisory group. In the first meeting we discussed and finalised the aims of the research given the data that was available. In the second meeting we discussed the research methods and what this could show us about how the people seeking advice may be changing as the rollout of UC continues. In the final meeting we discussed our findings, and how they related to the Citizens Advice advisors’ experiences of working with clients. We also discussed the wider policy landscape related to advice for UC and what this might mean for those who seek advice going forward.

We also discussed the interpretation of our results individually via Zoom with three people who had lived experience of seeking advice with claiming UC.

### Data

Citizens Advice collects data on clients’ demographic and health information for people in England and Wales during the advice seeking process. People may seek advice for more than one issue, we limit the data to those who are seeking advice with claiming UC. For in-person advice seeking, the information was collected by a self-completion paper questionnaire. For individuals seeking advice by phone or through a webchat, the questionnaire was administered verbally, and the answers were filled in by the Citizens Advice advisor.

The data is aggregated at a regional level. We know the number of observations by demographic profile, region and year (it is possible that people may seek advice more than once in a year with both UC and other issues). The dataset consists of 1,003,411 observations from all nine English Regions (East Midlands, Eastern, London, North East, North West, South East, South West, West Midlands, and Yorkshire & the Humber) and Wales for four financial years covering 1st April 2017 to 31st March 2021. We used 2017/18 to denote the financial year from 1st April 2017 to 31st March 2018. Similarly, we follow this pattern for the other three financial years: 2018/19, 2019/20, and 2020/21. Thus, we have data from before Citizens Advice was commissioned to provide advice by DWP (2017/18 to 2018/19) and after they were commissioned to provide advice (2019/20 to 2020/21). As an independent charity Citizens Advice were able to provide advice on the claim process before their DWP commission. However, after the commission claimants may have been signposted to Citizens Advice from the DWP if they needed additional support [11]. Data was collected on gender, age, ethnicity, disability, household type, housing tenure, employment, and marital status. Appendix Table A1 describes the data in more detail.

### Analysis

Descriptive statistics were presented to describe the socio-demographic and health characteristics of people seeking advice from Citizens Advice services in England and Wales over the period 2017/18-2020/21. First, we cleaned the data and explored missingness and how this may impact on our findings. Next, we summed the number of observations on people seeking advice with UC claims by year. Then, we calculated the proportion of observations for each variable by year. Finally, we employed a weighted t-test to understand the changes in socio-demographic characteristics of those seeking advice with Citizens Advice between each financial year (i.e. we compared 2017/18 to 2018/29 and 2018/29 to 2019/20 and 2019/20 to 2020/21). Specifically, we compared the changes in the number of people seeking advice in each demographic group with the changes in a reference group from the previous financial year. Weights are applied to the t-tests to account for the fact that each year in our study period more people are eligible for UC which will impact on the number of people seeking advice.

## Results

In Table 1, we presented a summary of the characteristics of people who seek advice with claiming UC from Citizens Advice between 2017 and 2021. We can see that the percentage of people seeking advice varies by region which is partially dependent upon the roll out of UC. Approximately 55% of claimants seeking advice were women for all years. Approximately 10% of the sample were aged below 25. People between 25 and 64 accounted for around 20% of the sample. For all years, more than 80% of the sample are of a white ethnic origin, which is similar to the 86% reported in the 2011 Census data [12] and the 85% reported in the 2019 ONS population estimates data [13]. Between 2017/18 and 2019/20, approximately 44% of the claimants reported a limiting long-term condition. In 2020/21, approximately 38% of people seeking advice reported a limiting long-term condition. The percentage of the claimants who reported a mental health condition was just over 29% in all years. This is higher than the UK average of approximately 25% [14]. Over the whole study period, more than one third of people seeking advice lived with dependent children, over half of the people seeking advice lived in council housing, 60% of the people seeking advice were unemployed and approximately 50% of the sample was single.

Missingness across the different variables are presented in Appendix A2. For all variables, as the number of people seeking advice increased over time, missingness for each variable also increased. The percent of missing data is high for some variables such as ethnicity and employment status. Data may not have been recorded or people may have preferred not to say. We cannot explore if missingness was systematic or random. However, it is possible that missingness may mean that our results are a lowerbound estimate of changes in proportions for different groups of people seeking advice.

Table 2 shows the changes in the proportion of people with the socio-demographic characteristics we are interested in between the financial years of (1) 2017/18 to 2018/19; (2) 2018/19 to 2019/20; (3) 2019/20 to 2020/21 and if these changes were statistically significant between the study years. 95% confidence intervals are shown. During the study period the majority of people making a claim for UC would be those with legacy benefits who had a significant change in circumstances (natural migration) or who were making a new claim for UC and had not been receiving benefits before. The varying pace of UC delivery rollout across the country meant that by the end of 2018, the proportion of households claiming UC varied by area [16]. Over our study period, as more people moved from legacy benefits we hypothesise that the number of people in employment, those without children, those who were married or cohabiting, and those living in either the private rented sector or home owners would increase. Subsequently, we hypothesise that more of these people would seek advice. Alternatively, during our study period, we would expect less people with limiting long term conditions to be making claims as they would remain on legacy benefits. There is evidence that work capability assessments may not adequately capture poor mental health [17–20]; thus, a priori it is difficult to predict how the percentage of people with poor mental health may change over time.

In relation to our hypotheses we found when comparing 2017/18 to 2018/19, (2.40%, 95%CI: 1.31-3.50%) there were significantly higher proportion of people with limiting long term conditions seeking advice with claiming UC than those without. This trend changed between 2018/19 and 2019/20 and between 2019/20 and 2020/21 there was a statistically significant decrease in the proportion of people seeking advice who reported a limiting long-term condition (-6.75%, 95%CI: -9.62%--3.88%; and − 2.09%, 95%CI: -2.54%--1.64%) compared with those who did not in 2019/20 and 2020/21 respectively. The proportion of people seeking advice who reported a mental health condition did not statistically significantly change over the study period.

Compared to the proportion of people seeking advice without dependent children, the proportion of people seeking advice with dependent children increased by 5.51% (95%CI: 3.35-7.66%) between 2017/18 and 2018/19. However, there was no significant difference between the proportion of those with and without children seeking advice between 2018/19 and 2019/20 and 2019/20 to 2020/21. In terms of housing tenure, there was a statistically significant increase in the proportion of people seeking advice for those who owned a property compared to those who lived in council housing in 2018/19 (3.55%, 95%CI:1.55-5.54%) and 2020/21 (3.19%, 95%CI: 1.66-4.73%). In 2020/21, there was also a statistically significant increase in the proportion of people living in private rental accommodation compared to those who lived in council housing seeking advice (1.17%, 95%CI: 0.65-1.68%).

Compared to the those seeking advice who were unemployed, the proportion of those seeking advice who were employed or self-employed statistically significant increase between 2017/18 and 2018/19 (4.10%, 95%CI: 1.71-6.48%; 4.60%, 95%CI: 1.22-7.97%; and 5.64%, 95%CI: 3.79-7.49%). There was no significant change in proportion of employed and self-employed people seeking advice compared to those who were unemployed between 2018/19 and 2019/20. However, there was a statistically significant increase in the proportion of those who were employed (2.53%, 95%CI: 1.50-3.56%) and self-employed (2.26%, 95%CI: 1.29-3.23%) seeking advice on claiming UC between 2019/20 and 2020/21 compared to those who were unemployed. Compared to the proportion of people seeking advice who were single, there were statistically significant increases in the proportion of people seeking advice who were married or cohabiting across all years of the survey (2017/18 to 2018/19 5.01%, 95%CI;: 3.24-6.78%; 2018/19 to 2019/20: 4.88%, 95%CI: 2.47-7.29%,; and 2019/20: (0.64%, 95%CI: 0.38-0.91%).

Results from other variables can be seen in Table 2.

**Table 1:** The socio-demographic characteristics of people seeking advice with claiming UC by year

		2017/18			2018/19			2019/20			2020/21	
		No. of Claimants	% of Claimants		No. of Claimants	% of Claimants		No. of Claimants	% of Claimants		No. of Claimants	% of Claimants
		(1)	(2)		(3)	(4)		(5)	(6)		(7)	(8)
**Total Number of Claimants**		59,514			146,109			397,892			399,896	
**Region**												
East Midlands		3,343	5.62%		10,860	7.43%		29,974	7.53%		28,582	7.15%
East of England		6,154	10.34%		15,892	10.88%		40,368	10.15%		42,413	10.61%
London		6,018	10.11%		14,822	10.14%		47,083	11.83%		49,409	12.36%
North East England		4,331	7.28%		10,571	7.24%		29,432	7.40%		32,481	8.12%
North West England		9,390	15.78%		19,119	13.09%		59,015	14.83%		58,946	14.74%
South East England		8,027	13.49%		22,619	15.48%		51,521	12.95%		48,505	12.13%
South West England		9,991	16.79%		18,801	12.87%		31,967	8.03%		30,217	7.56%
Wales		3,218	5.41%		9,990	6.84%		29,679	7.46%		32,310	8.08%
West Midlands		4,424	7.43%		13,400	9.17%		41,093	10.33%		39,823	9.96%
Yorkshire & the Humber		4,618	7.76%		10,035	6.87%		37,760	9.49%		37,210	9.30%
Total		59,514			146,109			397,892			399,896	
**Gender**												
Female		32,237	56.10%		83,436	59.16%		198,528	55.82%		191,598	58.73%
Male		25,222	43.90%		57,590	40.84%		157,136	44.18%		134,664	41.27%
Total		57,459			141,026			355,664			326,262	
**Age Group**												
<25		6,181	10.95%		14,076	10.05%		31,247	8.69%		32,839	9.67%
25–34		11,968	21.20%		29,505	21.07%		74,120	20.61%		77,726	22.88%
35–44		11,496	20.36%		29,674	21.19%		79,556	22.12%		75,721	22.29%
45–54		13,722	24.31%		33,319	23.80%		81,397	22.63%		70,703	20.81%
55–64		12,013	21.28%		31,090	22.21%		80,261	22.32%		68,760	20.24%
> 64		1,077	1.91%		2,344	1.67%		13,054	3.63%		13,971	4.11%
Total		56,457			140,008			359,635			339,720	
**Ethnicity**												
Black/Asian/Mixed		6,984	13.12%		17,244	14.80%		52,373	19.28%		43,455	18.57%
White		46,242	86.88%		99,307	85.20%		219,246	80.72%		190,606	81.43%
Total		53,226			116,551			271,619			234,061	
**Limiting Long-term Conditions**												
Yes		24,453	47.89%		61,135	49.96%		146,295	44.32%		121,769	38.86%
No		26,609	52.11%		61,224	50.04%		183,810	55.68%		191,582	61.14%
Total		51,062			122,359			330,105			313,351	
**Mental Health Conditions**												
Yes		5,387	29.52%		15,033	29.03%		35,082	29.13%		30,820	29.26%
No		12,860	70.48%		36,758	70.97%		85,370	70.87%		74,509	70.74%
Total		18,247			51,791			120,452			105,329	
**Household Type**												
With Dependent Children		13,189	32.99%		32,866	37.51%		77,752	38.24%		68,250	38.76%
Without Dependent Children		26,793	67.01%		54,742	62.49%		129,456	63.67%		107,855	61.24%
Total		39,982			87,608			203,327			176,105	
**Housing Tenure**												
Owned/Mortgaged		5,759	14.52%		14,167	16.23%		33,197	16.44%		35,551	20.53%
Private Tenant		11,487	28.96%		25,493	29.21%		59,256	29.35%		52,943	30.57%
Council Tenant		21,345	53.81%		45,427	52.06%		103,710	51.36%		80,971	46.76%
Homeless		1,073	2.71%		2,179	2.50%		5,748	2.85%		3,714	2.14%
Total		39,664			87,266			201,911			173,179	
**Employment Status**												
Employed PT		4,161	13.63%		11,374	15.03%		27,515	15.17%		23,327	14.72%
Employed FT		4,166	13.65%		11,562	15.28%		26,121	14.40%		27,701	17.48%
Self-Employed		1,270	4.16%		3,613	4.78%		9,134	5.04%		9,424	5.95%
Unemployed		20,924	68.56%		49,108	64.91%		118,589	65.39%		98,000	61.85%
Total		30,521			75,657			181,359			158,452	
**Marital Status**												
Married/Cohabiting		10,008	26.54%		24,142	29.90%		63,408	34.15%		56,165	36.41%
Divorced/ Separated		5,471	14.51%		12,200	15.11%		24,805	13.36%		18,259	11.84%
Widowed		1,020	2.70%		2,279	2.82%		4,812	2.59%		3,547	2.30%
Single		21,216	56.25%		42,113	52.16%		92,653	49.90%		76,281	49.45%
Total		37,715			80,734			185,678			154,252	

*Note*: This table summarises the yearly number (#) and percentage (%) of UC claimants by socio-demographic and health characteristics from 2017 to 2020. The number and percentage of missing are shown in Appendix Table A2.

(1) PT refers part time. (2) FT refers full time.

**Table 2:** Changes in the proportion of people by socio-demographic characteristics between financial years 2017/18 to 2018/19, 2018/19 to 2019/20 and 2019/20 to 2020/21

		Change between 2017/18 to 2018/19				Change between 2018/19 to 2019/20			Change between 2019/20 to 2020/21	
		Change		95% CI		Change	95% CI		Change	95% CI
		(1)		(2)		(3)	(4)		(5)	(6)
**Gender**										
Male		Reference		-		Reference	-		Reference	-
Female		3.34%		1.71–4.98%		-3.65%	-5.48% to -1.83%		1.07%	0.67–1.47%
**Age Group**										
<25		Reference		-		Reference	-		Reference	-
25–34		2.28%		1.20–3.36%		2.45%	0.37–4.52%		-0.10%	-0.63–0.43%
35–44		3.53%		2.22–4.83%		4.19%	1.27–7.11%		-1.09%	-1.95% to -0.23%
45–54		1.68%		0.59–2.77%		1.64%	-0.55–3.84%		-1.93%	-2.94% to -0.92%
55–64		3.08%		1.58–4.59%		3.05%	0.77–5.34%		-2.05%	-3.12% to -0.98%
> 64		-1.14%		-3.84–1.56%		40.12%	26.56–53.68%		0.15%	-1.06–1.37%
**Ethnicity**										
White		Reference		-		Reference	-		Reference	-
Black/Asian/Mixed		4.08%		0.68–7.49%		10.07%	3.82–16.32%		0.23%	-1.80–2.27%
**Limiting Long-term Conditions**										
No		Reference		-		Reference	-		Reference	-
Yes		2.40%		1.31–3.50		-6.75%	-9.62% to -3.88%		-2.09%	-2.54% to -1.64%
**Mental Health Conditions**										
No		Reference		-		Reference	-		Reference	-
Yes		-0.45%		-1.88–0.98%		0.27%	-1.01–1.55%		0.08%	-0.48–0.63%
**Household Type**										
Without Dependent Children		Reference		-		Reference	-		Reference	-
With Dependent Children		5.51%		3.35%% to 7.66%		0.99%	0.01–1.98%		0.19%	-0.24–0.61%
**Housing Tenure**										
Council Tenant		Reference		-		Reference	-		Reference	-
Owned/Mortgaged		3.55%		1.55–5.54%		1.30%	-1.09–3.69%		3.19%	1.66–4.73%
Private Tenant		1.51%		-0.35–3.36%		0.88%	-0.57–2.33%		1.17%	0.65–1.68%
Homeless		-0.27%		-3.28–2.74%		5.81%	1.20–10.42%		-1.23%	-1.88% to -0.57%
**Employment Status**										
Unemployed		Reference		-		Reference	-		Reference	-
Employed PT		4.10%		1.71–6.48%		0.29%	-1.02–1.61%		0.28%	-0.22–0.77%
Employed FT		4.60%		1.22–7.97%		-1.21%	-2.39% to -0.04%		2.53%	1.50–3.56%
Self-Employed		5.64%		3.79–7.49%		2.37%	0.23–4.51%		2.26%	1.29–3.23%
**Marital Status**										
Single		Reference		-		Reference	-		Reference	-
Married/Cohabiting		5.01%		3.24–6.78%		4.88%	2.47–7.29%		0.64%	0.38–0.91%
Divorced/ Separated		2.59%		-1.25–3.94%		-1.51%	-2.79% to -0.24		-0.78%	-1.14% to -0.41%
Widowed		3.01%		0.71–5.31%		-0.81%	-2.15–5.24%		-0.82%	-1.19% to -0.45%

*Note*: This table shows the socio-demographic changes in the cohort of UC claimants who seek advice from CA by year. For example, in column (1), the change of 3.34% in female means, compared to men, the number of women claimants who seek advice from CA has increased by 3.34% from 2017/18 to 2018/19.

## Discussion

We co-produced an analysis of Citizens Advice data from 2017 to 2021 on all people who sought advice making a UC claim in England and Wales, and interpreted the findings with service users. We found that as more people become eligible for UC the composition of people seeking advice changed. Over our study period, there was a significant increase in the proportion of employed and self-employed people seeking advice compared to those who were unemployed. There was a significant decrease in the proportion of people with limiting long term conditions seeking advice compared to those without. We did not find any significant change in the proportion of people with mental health conditions seeking advice. We also found an increase in the proportion of people who owned or had a mortgage on their home seeking advice compared to those in council housing and those who were married or cohabiting compared to those who were single. These findings provide some support to our hypotheses but not all of our predictions are borne out in the data.

Understanding who is seeking advice with claiming UC and how this changes as UC is rolled out eventually to everyone in receipt of means tested social security benefits has important public health implications. The social security system is a key positive structural determinant of health [21]. However, if the system unintentionally creates barriers to access via complicated or hard to use claim processes this will have negative impacts on health via stress associated with both the claim process and reduction in resources [10]. This will then contribute to health inequalities which depending upon who is affected may also impact on future generations. The long-term consequences of this would be higher costs to the health service and reduced economic productivity. Thus, to ensure that the claim process is working effectively; it is essential to understand who is seeking advice and how this may be changing over time. The findings from this research can be used to inform service delivery planning to both the DWP and Citizens Advice.

To put our results into context we discussed our findings with staff from Citizens Advice local offices in the North East of England (Northumberland, Gateshead, Newcastle, and Sunderland) and three service users. The staff and volunteers from Citizens Advice highlighted the continuing rise in numbers of people seeking advice as UC is rolled out to more people. Staff mentioned how this increase in the number of people over all may explain the observed decrease in the proportion of people with limiting long term conditions seeking advice. This provides some support for hypothesis one that the rollout and overall increase in numbers contributed to a rising number of people seeking advice. This is consistent with a report by Citizens Advice [6] which found the DWP to react slowly to feedback from users on the claim process. Advisors highlighted other reasons why from all branches discussed how year on year their caseload is rising. Deteriorating economic conditions in the UK from both global and domestic factors has meant more people need to apply for UC. The Covid-19 pandemic had a big impact on the demographics of people accessing support from Citizens Advice due to furlough, redundancy, and closed businesses for those who are self-employed. There was a pause in the move from legacy benefits and a relaxation of some of the conditionality measures during the height of the Covid-19 pandemic (March 2020-September 2021) [22] which may have impacted on the composition of people seeking advice potentially contributing to the reduction in the proportion of those with limiting long term health conditions seeking advice. This is in support of hypothesis 2.

From the 1st April 2022, DWP substantially reduce the funding it provided to the ‘Help to Claim’ Service. These changes in funding mean that not all local offices are now able to provide support for claiming with UC and Citizens Advice only receives funding to provide telephone and online support for UC claims. This will reduce the number of people that Citizens Advice can support with claiming UC. In addition, Help to Claim service is only funded until March 2023 and large numbers of clients will be moved to UC through the managed migration process. However, this process is not automatic. Clients will need to apply for UC and many will need support with this until December 2024 or their benefit payments will end. The need for the Help to Claim Service and especially face-to-face advice is needed and Citizens Advice is well placed to be able to provide this support. Nationally, Citizens Advice has highlighted in a recent report [6] ways to improve their relationship with the DWP to increase their capacity to support more people such as reducing the time between acknowledging issues and faults with the UC application process and making changes to these processes.

The service users we spoke to discussed the long wait times before being able to speak to a Citizens Advice advisor which had led to additional financial stress as it increased the time it took to make their claim and thus to receive their first payment. All service users mentioned that the claim process was confusing and involved jargon and a difficult to navigate webpage. This is consistent with findings from the literature that many people struggled with the claim process [5]. A recommendation from the service users was a clear flow chart explaining the process when claiming UC and where to get help and greater involvement of service users in the design of the website to claim UC.

There are some similarities but also some differences with the composition of people seeking advice in our data and data collected by the Department for Work and Pensions [13] which summarised data on all UC claimants from April 2013 to January 2021. In both datasets there was an increase in the proportion of men. However, in our dataset from 2018 on we did not find an increase in advice seeking for households without children. This suggests that people with children may be more likely to seek advice with making a claim and should be considered in service delivery planning going forward.

Having a service that is easy to use and providing people with the opportunity to seek advice in a timely format in a manner that supports their needs can help to reduce some of the negative health impacts of UC [5, 7–10, 15]; potentially helping to reduce growing health inequalities in the UK.

### Strengths and limitations

The strength of our study is that we co-designed this research with Citizens Advice using a unique large nationally representative dataset covering the period 2017–2021. This allowed us to conduct a comprehensive descriptive analysis which can help inform policy to improve the advice seeking process as the rollout of UC continues. We also discussed the research with service users to develop policy recommendations which can simplify the claim process, potentially reducing some of the stress and negative health impacts associated with UC.

There are several limitations. We are not able to identify people who have sought advice numerous times over a year. Due to the nature of the data, we were also not able to perform more complex quantitative analysis or draw any causal conclusions regarding how the advice process is associated with the health of UC claimants. Evidence does suggest that seeking advice can reduce stress and support coping [23]. We have not tested this here, but present our findings as cautiously building on these past conclusions from other studies. Having greater service user involvement in the research design and data analysis could have been beneficial in identifying recommendations.

## Electronic supplementary material

Below is the link to the electronic supplementary material.

Supplementary Material 1

## Data Availability

All data is publicly available and can be accessed from here: https://public.tableau.com/app/profile/citizensadvice.
