# Supplementary material for: Exploring the health and sociodemographic characteristics of people seeking advice with claiming universal credit: a cross-sectional analysis of UK citizens advice data, 2017–2021
Source: BMC Public Health. 2023 Mar 30;23:595. doi: 10.1186/s12889-023-15483-4 (PMC10060933; doi:10.1186/s12889-023-15483-4)
Supplement: Supplementary file 1 — Supplementary Material 1 [file 12889_2023_15483_MOESM1_ESM.docx]

| **Table A1. List of socio-demographic and health characteristics** | |
| --- | --- |
|  | **Sub-Groups** |
| **Claim Subjects** | 01 (Initial Claim); 02 (Income Support, Standard elements); 03 (Housing elements); 04 (Disability elements, Limited capability for work); 05 (Child elements); 06 (Childcare costs); 07 (Carer elements, Pay & Entitlements); 08 (Calculation of income and capital); 09 (Conditionality and Commitment); 10 (Universal Support); 11 (Deductions); Other/N.A. |
| **Gender** | Female; Male; Other/Not Given |
| **Age** | ≤19; 20-24; 25-29; 30-34; 35-39; 40-44; 45-49; 50-54; 55-59; 60-64; ≥65; Other/Not Given |
| **Ethnicity^1^** | Asian; Black; Mixed; White; Other/Not Given |
| **Disability^1^** | Long-Term Health Condition (LTH); Mental Health; Other/Not Given |
| **Household Type** | Couple; Couple with Dependent Children; Other Adults; Other Adults with Dependent Children; Single; Single with Dependent Children; Other/Not Given |
| **Housing Tenure** | Buying Home (Mortgage, etc); Council Tenant; Homeless; Own Outright or Shared Ownership; Private Tenant; Other/Not Given |
| **Employment Status** | Carer; Employed < 16hrs p/w; Employed between 16-29hrs p/w; Employed ≥ 30hrs p/w; Retired; Self-Employed; Student; Unpaid Work; Other/Not Given |
| **Marital Status** | Married/Cohabiting/Civil Partnership; Divorced; Separated but Legally Married/CP; Single; Widowed/Surviving CP partner; Other/Not Given |
| *Note:* 1. Ethnicity and Disability status are self-reported | |

**Appendix**

| **Table A2. Missing observations for the England and Wales data by year** | | | | |
| --- | --- | --- | --- | --- |
|  | **2017** | **2018** | **2019** | **2020** |
|  | **(1)** | **(2)** | **(3)** | **(4)** |
| **Number of Claimants** | 59,514 | 146,109 | 397,892 | 399,896 |
| **Gender** |  |  |  |  |
| # of Missing | 2,055 | 5,083 | 42,228 | 73,634 |
| % of Missing | 3.45% | 3.48% | 10.61% | 18.41% |
| **Age Group** |  |  |  |  |
| # of Missing | 3,057 | 6,101 | 38,257 | 60,176 |
| % of Missing | 5.14% | 4.18% | 9.61% | 15.05% |
| **Ethnicity** |  |  |  |  |
| # of Missing | 6,288 | 29,558 | 126,273 | 165,835 |
| % of Missing | 10.57% | 20.23% | 31.74% | 41.47% |
| **Limiting Long-term Conditions** |  |  |  |  |
| # of Missing | 8,452 | 23,750 | 67,787 | 86,545 |
| % of Missing | 14.20% | 16.25% | 17.04% | 21.64% |
| **Mental Health Conditions** |  |  |  |  |
| # of Missing | 41,267 | 94,318 | 277,440 | 294,567 |
| % of Missing | 69.34% | 64.55% | 69.73% | 73.66% |
| **Household Type** |  |  |  |  |
| # of Missing | 19,532 | 58,501 | 194,565 | 223,791 |
| % of Missing | 32.82% | 40.04% | 48.90% | 55.96% |
| **Housing Tenure** |  |  |  |  |
| # of Missing | 19,850 | 58,843 | 195,981 | 226,717 |
| % of Missing | 33.35% | 40.27% | 49.25% | 56.69% |
| **Employment Status** |  |  |  |  |
| # of Missing | 28,993 | 70,452 | 216,533 | 241,444 |
| % of Missing | 48.72% | 48.22% | 54.42% | 60.38% |
| **Marital Status** |  |  |  |  |
| # of Missing | 21,799 | 65,375 | 212,214 | 245,644 |
| % of Missing | 36.63% | 44.74% | 53.33% | 61.43% |
| *Note:* This table shows the number (#) and percentage (%) of missing observations for the England and Wales data by year. The % of missing is calculated by the # of missing over the total number of observations of those seeking advice | | | | |
